# Focus on Filgotinib in Rheumatoid Arthritis: A Trial-Based Review

**DOI:** 10.31138/mjr.281123.fof

**Published:** 2024-03-30

**Authors:** Elpida Skouvaklidou, Dimitrios Deligeorgakis, Anastasia Skalkou, Vasileios Skepastianos, Konstantinos Tsafis, Evdokia Papadimitriou, Eleni Pagkopoulou, Paraskevi Avgerou, Maria G Mytilinaiou, Maria Tzitiridou-Chatzopoulou, Nikolaos Kougkas, Christina Adamichou

**Affiliations:** 1 Department of Rheumatology, 4th Department of Internal Medicine, Hippokration Hospital, Thessaloniki, Greece,; 2 School of Healthcare Sciences, Department of Midwifery, University of Western Macedonia, Kozani, Greece

**Keywords:** Janus kinases, filgotinib, rheumatoid arthritis, randomised clinical trials, efficacy, safety

## Abstract

Janus kinases (JAK)/Signal Transducer and Activator of Transcription (STAT) pathway is involved in pathophysiologic cascade of a notable number of rheumatic diseases. The development of JAK inhibitors has expanded treatment choices in rheumatoid arthritis (RA) with a sustained class-effect efficacy. Filgotinib is a novel selective inhibitor of JAK1 isoform licensed for use in RA and ulcerative colitis. In this review we aim to present an analysis of filgotinib’s efficacy and drug-specific safety warnings. Patients with RA with or without concomitant conventional synthetic Disease-Modifying Antirheumatic Drugs (csDMARDs) (naïve or experienced) and those who have failed biologic Disease-Modifying Antirheumatic Drugs (bDMARDs) were examined in randomised clinical trials. Filgotinib was also tested against placebo, methotrexate, or adalimumab. Long-term extension trials provide insights for up to four years of continuous filgotinib administration. Beneficial effects are depicted in both disease activity parameters and quality of life indexes in moderate or severe RA with a longitudinal efficacy. In head-to-head comparison with adalimumab, filgotinib 200 mg was non-inferior. Adverse effects alerts are marked by the elevated risk of infectious adverse effects with the exception of herpes zoster infection, which has a low incidence.

## INTRODUCTION

Janus kinases (JAKs) are immunomodulatory mediators involved in the JAK/signal transducer and activator of transcription (STAT) pathway and participate in autoimmune and inflammatory cytosolic and nucleic signals in close synergy with cytokines and cytokines receptors.^1^ Semantically, their name derives from the roman god Janus identified as the god of beginnings or doorways implying their key role in autoimmune rheumatic diseases.

The JAK/STAT pathway is activated by extracellular signalling molecules (cytokines) which bind to the outer domain of a membrane receptor. These receptors also possess an intracellular domain who acts as an anchor for JAKs docking. Once cytokines link to the receptors the associated JAKs are activated and phosphorylate tyrosine residues on the receptor which then recruits STAT proteins. When employed, the STAT proteins act as transcription factors, migrate to the nucleus and modulate specific genes. Cytokines binds to their respective receptors with great selectivity which are also uniquely associated with a pair of JAKs. These pairs are formed through combinations of the four members of JAK family, namely JAK1, JAK2, JAK3, and tyrosine kinase 2 (TYK2).^2^

Several known disease-specific cytokines exert their pathophysiological actions through JAK/STAT mechanisms; interleukin (IL)-6, involved in the development of rheumatoid arthritis (RA), is associated with JAK1, JAK2 and TYK2^3^ while IL-12/IL-23, implicated in the pathogenesis of spondylarthritis (SpA) spectrum, binds to JAK2 and TYK2.^2,4^

The discovery of the pathogenetic role of JAK/STAT pathway led to the development of JAK inhibitors (JAKi) with the intention to block the pathophysiologic cascade and effectively treat patients with inflammatory diseases. This new orally available drug class of targeted synthetic Disease-modifying antirheumatic drugs (ts-DMARDs) enumerates already four European Medicines Agency (EMA) approved molecules with different JAK selectivity and distinct indications and precautions.^5–8^ Tofacitinib was the first JAK1, JAK2, and JAK3 inhibitor discovered, and is currently approved for treatment of inflammatory arthritides [RA, ankylosing spondylitis (AS), psoriatic arthritis (PsA), juvenile idiopathic arthritis] and ulcerative colitis (UC).^7,9^ Baricitinib mitigates arthritic symptomatology for patients with rheumatoid arthritis by inhibiting JAK1 and JAK2,^10^ and is also licensed for atopic dermatitis and alopecia areata.^6^ The third JAK inhibitor developed was upadacitinib, which selectively inhibits JAK1 isoform contrary to tofacitinib and baricitinib.^11^ Upadacitinib is an authorised therapeutic option for patients with RA, PsA, axial SpA, UC, and atopic dermatitis.^8^

The newest tsDMARD with eclectic JAK1 inhibitory mode of action is filgotinib (FIL). This molecule proved efficacious in patients with RA and UC which enabled its EMA approval in 2020.^5^ In this article we comprehensively review current clinical trial data and evaluate the use of FIL for its presently authorized indications with particular emphasis in rheumatoid arthritis.

## FILGOTINIB IN RHEUMATOID ARTHRITIS

### Effectiveness in phase II/III trials

#### Filgotinib in methotrexate-resistant RA

Primary studies to investigate FIL’s effectiveness in RA were conducted in patients who could not achieve low disease activity or disease remission with methotrexate (MTX). DARWIN 1, a phase IIb, 24-week, randomized clinical trial (RCT) divided 594 patients into six FIL treated groups (50/100/200 mg daily or 25/50/100 mg twice daily) and one placebo group to establish efficacious dosage (**Table 1**).^12^ A significant difference was observed in the American College of Rheumatology (ACR)20 response (12 weeks) in FIL 200 mg daily, 100 mg daily and 100 mg twice daily treated arms versus placebo. For ACR 20/50/70 responses, the higher the FIL dose, the highest the number of patients achieving these responses was. All FIL-treated arms at 24 weeks mastered a greater ACR50, ACR70 and Health Assessment Questionnaire-Disability Index (HAQ-DI) in comparison to placebo arm. (all p<0.05). At week 12, 66 patients either on placebo or on FIL <100 mg total daily dose who did not achieve an ACR20 response, were reassigned to receive 100 mg total daily dose, and achieved better ACR20/50/70 responses. Both clinical assessment scores, Disease Activity Score (DAS) 28 [C-reactive protein (CRP)] and Clinical Disease Activity Index (CDAI) showed a significant dose-response decrease in all FIL treated patients (except 50 mg FIL in CDAI) at week 24. This dose-response relationship depicted in DARWIN 1 established the efficacy of 100 mg and 200 mg FIL doses over lower ones.

**Table 1.** Characteristics of filgotinib’s rheumatoid arthritis trials.

**Investigators Type Trial number Name**	**Diagnosis**	**Disease characteristics**	**Number of Patients randomized**		**Intervention**	**Intervention dose/time of administration**	**Duration of intervention**	**Co-medication (DMARDs)**	**Primary endpoints**	**Secondary endpoints**
Westhovens et al NCT01888874 RCT phase IIb DARWIN 1	RA	Moderate-to-severe activity, bDMARDs naive	594 (1:1:1:1:1:1:1) Placebo		FIL	50/100/200 mg once daily or 25/50/100 mg twice daily	24 weeks	MTX (stable dose)	ACR20 (12 weeks)	ACR50/70, ACR-N, DAS28 (CRP), LDA/ remission, EULAR response, ACR/EULAR remission CDAI, SDAI HRQoL HAQ-DI
					Twice daily					
Kavanaugh et al RCT phase II b NCT 01894516 DARWIN 2	RA	Moderate-to- severe activity, MTX failure	283 (1:1:1:1) Placebo		FIL	50/100/200 mg daily	24 weeks	None (≥4-week washout from MTX)	ACR20 (12 weeks)	ACR20/50/70, ACR-N, DAS28 (CRP), LDA/ remission, EULAR response, ACR/EULAR remission CDAI, SDAI, HRQoL, HAQ-DI
					Capsules daily					
Kavanaugh et al. Open LTE study NCT02065700 DARWIN 3	RA	LTE study of DARWIN 1,2	739	497	FIL	200 mg once or 100 mg twice daily	4 years	MTX	Adverse effects, laboratory abnormalities	ACR20/50/70 DAS28 (CRP)
				242		200mg once or 100mg twice daily		None		
Tanaka et al Phase III NCT02889796 FINCH 1	RA	Moderate-to-severe activity	147	40	FIL	200 mg daily	24 weeks	MTX (stable dose)	ACR20 (12 weeks)	ACR50/70, DAS28 (CRP) HAQ-DI, SF-36, PCS, FACIT, SJC, TJC, pain, PGA (physician’s and patient’s), hsCRP
				41	FIL	100 mg daily				
				28	ADA	40 mg biweekly				
				48	Placebo	Tablets or injections				
Genovese et al Phase III NCT02873936 FINCH 2	RA	Moderate-to- severe RA, inadequate response/ intolerance to ≥1 prior bDMARDs	449 (1:1:1) FIL Placebo		FIL	200 mg daily	24 weeks 100% (83%) MTX 99.3% (78.4% MTX)	100% (84.4% MTX)	ACR20 (12 weeks)	ACR 20/50/70, DAS28(CRP), HAQ-DI, SF-36, FACIT, SDAI, CDAI
					100 mg daily					
					Tablets daily					
Westhovens et al RCT Phase III NCT 02886728 FINCH 3	RA	MTX naïve patients	1252 (2:1:1:2) FIL FIL MTX		FIL	200 mg daily	52 weeks 100% None None	100%	ACR20 (24 weeks)	ACR 20/50/70, HAQ- DI, DAS28(CRP), mTSS, SF-36 PCS, FACIT-F, SDAI, CDAI, PAIN, SJC, TJC, pain, PGA (physician’s and patient’s), hsCRP
					100 mg daily					
					200 mg daily					
					Orally once weekly					
Tanaka et al. LTE study NCT03025308 FINCH 4	RA	LTE study of FINCH 1 eligible patients	115 (56/59)		FIL 100 mg daily	200 mg daily	48 weeks	MTX (stable dose)	Adverse effects, laboratory abnormalities	ACR 20/50/70, DAS28(CRP), CDAI, SDAI, and Boolean remission endpoints, SF-36 PCS, FACIT- Fatigue, HAQ-DI, pain, hsCRP
Combe et al RCT phase III NCT02889796	RA	Moderate-to-severe activity	1755 (3:3:2:3) FIL ADA Placebo		FIL	200 mg daily	52 weeks	MTX (stable dose)	ACR20 (week 12)	HAQ-DI, DAS28(CRP), Radiographic progression (mTSS), SF-36, FACIT-F, ACR 50/70, SDAI, CDAI
					100 mg daily					
					40 mg biweekly					
					Tablets daily or injections	24 weeks				

ACR; American College of Rheumatology: ADA; Adalimumab: bDMARDs; biologic Disease-Modifying Antirheumatic Drugs: CDAI; Clinical disease activity index: CRP; C-reactive protein: DAS; Disease activity score: EULAR; European Alliance of Associations for Rheumatology: FACIT-F; Functional Assessment of Chronic Illness Therapy Fatigue Scale: FIL; Filgotinib: HAQ-DI; Health Assessment Questionnaire-Disability Index: HRQol; Health-Related Quality of Life: LDA; Low disease activity: LTE; long-term extension: MTX; Methotrexate: mTSS; modified Total Sharp Score: NCT; National Clinical Trials: PGA; patient or physician global assessment: RA; Rheumatoid arthritis: RCT; Randomised controlled trial: SDAI; Simplified disease activity index: SF-36 PCS; Short Form 36–Physical Component Score: SJC; Swollen joint count: TJC; Tender joint count

Another RCT explored the impact of FIL in disease activity parameters in patients with moderately or severely active RA already under treatment with MTX. Combe et al. in their phase III trial randomised patients into four treatment groups, FIL 100 mg daily, FIL 200 mg daily, Adalimumab (ADA) 40 mg biweekly or placebo for 52 weeks (**Table 1**).^13^ FINCH 1 trial (phase III RCT) assessed the Japanese sub-population from the original pool of 1755 patients of Combe et al up to 24 weeks (**Table 1**).^14^ For the original population, primary endpoint, ACR20 response, was met for significantly more patients in the FIL plus MTX groups versus the placebo plus MTX group [76.6% for FIL 200 mg and 69.8% for FIL 100 mg versus 49.9% for placebo (all p<0.001)]. Key secondary points, HAQ-DI and DAS28(CRP) <2.6, also performed significantly better for FIL compared to placebo at 12 weeks. At 24 weeks radiographic measurements were favourable for the FIL groups (p<0.05). Concerning head-to-head comparison with ADA, only FIL 200 mg was non-inferior for DAS28(CRP) ≤3.2 at 12 weeks.^13^ The Japanese sub-population had consistent results with the original study population for the primary and key secondary outcomes.^14^

#### Filgotinib in unresponsive/intolerant to bDMARDs RA

FINCH 2 study uniquely explored the effects of FIL in patients with moderate to severe RA who were refractory to one or more biologic disease-modifying antirheumatic drugs (bDMARDs), receiving concomitant stable treatment with csDMARDs (**Table 1**).^15^ This phase III RCT included 449 patients, randomised to receive FIL 200 mg, FIL 100 mg or placebo for 24 weeks. Primary endpoint, ACR20, was met for 66.0% and 57.5% of FIL, 200 mg and 100 mg, respectively, and 31.1% of placebo treated patients, proving a significant difference. Furthermore, key secondary outcomes, namely DAS28(CRP) (<2.6 and <3.2) and HAQ-DI presented significant superiority for FIL group as compared to placebo at 12 and 24 weeks.

#### Filgotinib in MTX-naïve RA

In FINCH 3 study, a phase III RCT, FIL (100 mg or 200 mg) plus MTX outweighed MTX monotherapy in MTX naïve patients when ACR20/50/70, DAS28(CRP)<2.6 and HAQ-DI responses were considered (all p<0.05) at 24 weeks (**Table 1**).^16^ Furthermore, patients on any FIL dose plus MTX performed better on Short Form 36–Physical Component Score (SF-36 PCS) and had less radiographic progression versus patients on MTX alone, while Functional Assessment of Chronic Illness Therapy Fatigue Scale (FACIT-F) scores were interchangeable.

#### Filgotinib monotherapy in RA

FIL as stand-alone, once daily, 24-weeks therapy was firstly explored in a phase II RCT with three actively treated and a placebo arm, the DARWIN 2 study (**Table 1**).^17^ DARWIN 2 recruited 283 patients with moderate to severe active RA who had failed MTX treatment and underwent a minimum of four weeks wash out period prior to study initiation. Primary outcome was ACR20 response to treatment at week 12, which was achieved in all FIL arms (50 mg, 67%; 100 mg, 66%; 200 mg, 73%) in comparison to placebo (29% all p<0.0001). A similar pattern was followed by ACR50 responses at 12 weeks for FIL-treated patients, while patients receiving the highest FIL doses were more likely to reach an ACR70 response. Placebo and FIL 50 mg treated patients who failed to reach the ACR20 goal were re-randomised to FIL 100 mg at week 12 and exhibited a similar ACR20/50/70 response to the original 100 mg arm at week 24. The proportion of patients with ACR20/50 responses was sustained throughout the study, and even increased in the case of ACR70. DAS28(CRP), CDAI, Simplified disease activity index (SDAI), EULAR "good" responses and Health-Related Quality of Life (HRQoL) significantly improved in all FIL arms at week 12 compared to placebo. DARWIN 2 displayed that FIL dose and disease activity were generally inversely related. Westhovens et al. in their phase III RCT randomised 1252 RA patients with limited or no MTX exposure to receive either FIL (in two distinct doses) in combination with MTX or mono-therapy with FIL/methotrexate (FINCH 3 study, **Table 1**).^16^ For the ACR20 response, FIL 200 mg monotherapy when compared to methotrexate alone showed no superiority at week 24, contrary to week 52 when FIL significantly prevailed. Furthermore, comparison in both arms for SF-36 and FACIT-F proved neutral, however they differed significantly in modified Total Sharp Score (mTSS), HAQ-DI in favor of FIL and a higher number of patients receiving FIL achieved a DAS28(CRP)<2.6 and ACR50/70 responses at weeks 24 and 52.^16^

### Long-term extension studies

Useful insights into FIL’s effectiveness and safety in the long run, are provided by long term extension studies (LTE). DARWIN 3 (**Table 1**) study recruited eligible patients from DARWIN 1 and 2 for an additional period of four years, while they received treatment with FIL 200 mg total daily dose with or without MTX. This trial conferred results emphasising FIL’s longitudinal benefit in RA activity scores with an ACR20/50/70 response of 89.3%/69.6%/49.1% and 91.8%/69.4%/44.4% in the FIL plus MTX and FIL monotherapy groups respectively. By the study end, approximately half of the patients on any group achieved a DAS28(CRP)<2.6.^18^ However, DARWIN 3 study results were hindered by the high discontinuation rate (46.7% in FIL plus MTX group and 43.8% in FIL monotherapy group) mainly attributed to adverse effects and should be viewed with caution. FINCH 4 is the extension study of FINCH 1 with a currently ongoing horizon of 6 years follow-up (**Table 1**). Preliminary data up to 48 weeks are provided for the two group of patients studied (FIL 100 mg plus MTX, FIL 200 mg plus MTX) after rerandomising the ADA group from the parent study. At 48 weeks, 94% of patients in the FIL 200 mg plus MTX arm and 92% of FIL 100 mg plus MTX arm managed an ACR20 response. ACR 50/70 were 75%/57% and 83%/58% for FIL 200 mg plus MTX and FIL 100 mg plus MTX respectively. Disease activity measured as DAS28(CRP)<2.6 was reported for 81% of patients treated with FIL 200 mg and 74% of patients in the FIL 100 mg arm.^19^

### Safety

FIL proved to be well tolerated during DARWIN 1 and DARWIN 2 phase IIb trials with similar rate of adverse events reported across all study groups for each study.^12,17^ Serious infections occurred in 6 (one received placebo) and 4 (one received placebo) patients in DARWIN 1 and 2 respectively, while herpes zoster infection occurred in collectively 6 patients (one received placebo). DARWIN 1 reports two cardiovascular events namely a stroke and a myocardial infraction during study duration.^12^ Data from DARWIN 3 trial, which investigated adverse events for up to 4 years of FIL’s continued use, revealed a low exposure-adjusted incidence rates (EAIRs) per 100 patient-years of exposure (PYE) for any serious infection (0.6 in FIL plus MTX group and 1.7 in FIL monotherapy group). Similar low EAIRs are for herpes zoster infection (1.3 and 1.5 per 100 PYE in the FIL plus MTX and monotherapy groups, respectively) and major cardiovascular events (MACE) (0.2 per 100 PYE for both arms). For all infectious adverse effects combined in DARWIN 3, calculated EAIRs were 16.3 in the FIL plus MTX arm and 15.9 in FIL monotherapy arm.^18^ On the contrary, in the phase III trial performed by Combe et al., patients with RA treated with FIL or ADA experienced more infections and serious infections versus patients receiving placebo. Herpes zoster infection was reported in 0.4% of FIL or placebo treated patients and 0.6% of ADA treated. Furthermore, venous thromboembolism occurred in one patient from FIL group and two patients from placebo group and MACE occurred in four patients (one in FIL, two in ADA and one in placebo arm). All in all, integrated results from DARWIN 1–3 and FINCH 1–4 studies outline a higher rate of infectious adverse effects (apart from herpes zoster infection) among FIL treated patients versus placebo for the placebo-controlled time.^20^ Calculated EAIRs for deaths were similar for FIL and placebo (0.6/100PYE) while herpes zoster infections, MACE, and venous thromboembolism were infrequently reported.^20^

## FILGOTINIB IN OTHER INFLAMMATORY DISEASES

A limited number of studies examined FIL’s efficacy in mitigating arthritic symptomatology in patients with SpA. These studies were phase II RCTs and provided beneficial evidence. For AS in TORTUGA trial (**Table 2**), FIL provided a clear benefit, as patients on FIL achieved a significant reduction in Ankylosing Spondylitis Disease Activity Score (ASDAS) compared to placebo treated patients. Furthermore, a higher proportion of patients reached an Assessment of SpondyloArthritis international Society (ASAS) 20 and ASAS40 response in the FIL arm versus the placebo arm.^21^ On the other hand, EQUATOR trial was designed to test FIL’s potential as a therapeutic agent in patients with PsA (**Table 2**). Results show that more patients in the FIL arm achieved an ACR20/50/70 response compared with placebo and these were all significant differences. FIL also managed greater improvements in Disease Activity Index for Psoriatic Arthritis (DAPSA) scores and psoriasis [measured with Psoriasis Area and Severity Index 75(PASI75)] versus placebo.^22^ However, both TORTUGA trial and EQUATOR trial had a small number of participants, and more studies are needed to confirm these results.^21,22^

**Table 2.** Characteristics of filgotinib’s psoriatic arthritis and ankylosing spondylitis trials.

**Investigators Trial number Type Name**	**Diagnosis**	**Disease characteristics**	**Number of Patients randomized**	**Intervention**	**Intervention dose/time of administration**	**Duration of intervention**	**Co-medication (DMARDs)**	**Primary endpoints**	**Secondary endpoints**
van der Heijde et al RCT phase II NCT03117270 TORTUGA	AS	Active disease (BASDAI≥4) Spinal pain ≥4, Inadequate response, or intolerance to ≥2 NSAIDs	116 (1:1)	FIL	200 mg daily	12 weeks	40% csDMARDs,	ASDAS (change from baseline through 12 weeks)	ASDAS (change over time), ASAS20, ASAS40, ASAS5/6, ASAS partial remission, BASDAI, BASFI, BASMI, SJC, TJC, SPARCC MRI score, SF-36, ASQoL
				Placebo	Tablets daily		38% csDMARDs		
Mease et al RCT phase II NCT03101670 EQUATOR	PsA	Moderate-to- severe activity, insufficient response, or intolerance to ≥1 csDMARDs	131 (1:1)	FIL	200 mg	16 weeks	72% csDMARDs,	ACR20 (16 weeks)	ACR20/50/70, DAS28 (CRP), PsARC, SPARCC Enthesitis Index, PASI75, mNPSI, MDA, pruritus, HAQ-DI, pain, fatigue, FACIT-F, DAPSA, PASDAS, LEI
				Placebo	Capsules daily		76% csDMARDs		

ACR; American College of Rheumatology, AS; Ankylosing Spondylitis, ASAS; Assessment of Spondyloarthritis International Society, ASQoL; Ankylosing Spondylitis Quality of Life, ASDAS; Ankylosing Spondylitis Disease Activity Score, BASDAI; Bath Ankylosing Spondylitis Disease Activity Index, BASFI; Bath Ankylosing Spondylitis Functional Index, BASMI; Bath Ankylosing Spondylitis Metrology Index, csDMARDs; conventional synthetic Disease-Modifying Antirheumatic Drugs, DAS; Disease activity score, DAPSA; Disease Activity Index for Psoriatic Arthritis, FACIT-F; Functional Assessment of Chronic Illness Therapy Fatigue Scale, FIL; Filgotinib, HAQ-DI; Health Assessment Questionnaire-Disability Index, LEI; Leeds enthesitis index, MRI; Magnetic Resonance Imaging, MDA; Minimal Disease Activity, mNPSI; modified Nail Psoriasis Severity Index, NCT; National Clinical Trials, NSAIDs; Non-Steroidal Anti-Inflammatory Drugs, PASDAS; Psoriatic Arthritis Disease Activity Score, PASI; Psoriasis Area and Severity Index, PsA; Psoriatic Arthritis, PsARC; Psoriatic Arthritis Response Criteria, RCT; Randomised controlled trial, SF-36; Short Form 36, SJC; Swollen joint count, SPARCC; Spondyloarthritis Research Consortium of Canada, TJC; Tender joint count

Inflammatory bowel diseases are another therapeutic target for JAKi with tofacitinib and upadacitinib already being used successfully in UC.^7,8^ Gastroenterologists include FIL in their official armamentarium for the treatment of UC based on positive results from SELECTION and SELECTIONLTE trials.^23,24^ For Crohn’s disease, results were ambiguous; in DIVERGENCE trial FIL performed poorly and did not meet trial’s endpoints,^25^ while in FIRZROY trial more patients receiving FIL achieved disease remission compared to placebo.^26^

## CONCLUSION

Filgotinib is a selective inhibitor of JAK1 isoform and the latest of this class to receive official approval for use in rheumatoid arthritis. Beneficial effects are depicted in both disease activity parameters and quality of life indexes in moderate or severe RA with or without background csDMARDs and in patients who have failed bDMARDs. In head-to-head comparison with ADA, FIL 200 mg was non-inferior. LTE studies illustrate a longitudinal efficacy profile of filgotinib while safety profile is marked by the elevated risk of infectious adverse effects, with the exception of herpes zoster infection which has a low incidence.

## ABBREVIATIONS

ACR: American College of Rheumatology
ADA: Adalimumab
AS: Ankylosing spondylitis
ASAS: Assessment of SpondyloArthritis international Society
ASDAS: Ankylosing Spondylitis Disease Activity Score
bDMARDs: biologic Disease-Modifying Antirheumatic Drugs
CRP: C-Reactive Protein
CDAI: Clinical Disease Activity Index
csDMARDs: conventional synthetic Disease-Modifying Antirheumatic Drugs
DAPSA: Disease Activity Index for Psoriatic Arthritis
DAS: Disease Activity Score
EAIRs: Exposure-Adjusted Incidence Rates
EMA: European Medicines Agency
FACIT-F: Functional Assessment of Chronic Illness Therapy Fatigue Scale
FIL: Filgotinib
HAQ-DI: Health Assessment Questionnaire-Disability Index
HRQoL: Health-Related Quality of Life
IL: Interleukin
JAKi: Janus kinase inhibitors
JAKs: Janus kinases
LTE: Long-term extension studies
MACE: Major cardiovascular events
MTX: methotrexate
mTSS: modified Total Sharp Score
PsA: Psoriatic arthritis
PASI: Psoriasis Area and Severity Index
PYE: Patient-years of exposure
RA: Rheumatoid Arthritis
RCT: Randomised Clinical Trial
SDAI: Simplified disease activity index
SF-36 PCS: Short Form 36–Physical Component Score
SpA: Spondyloarthritis
STAT: Signal Transducer and Activator of Transcription
ts-DMARDs: targeted synthetic Disease-Modifying Antirheumatic Drugs
TYK2: Tyrosine kinase 2
UC: Ulcerative Colitis
